# Contrast-Induced Nephropathy: A Review of Mechanisms and Risks

**DOI:** 10.7759/cureus.14842

**Published:** 2021-05-04

**Authors:** Elham Shams, Harvey N Mayrovitz

**Affiliations:** 1 Osteopathic Medicine, Nova Southeastern University Dr. Kiran C. Patel College of Osteopathic Medicine, Davie, USA; 2 Medical Education, Nova Southeastern University Dr. Kiran C. Patel College of Allopathic Medicine, Davie, USA

**Keywords:** contrast media, contrast-induced nephropathy, pathophysiology, prevention, management, n-acetylcysteine, renal injury

## Abstract

Radiological procedures utilizing intravenous iodinated contrast agents are being widely utilized for both therapeutic and diagnostic purposes. This has resulted in an increasing incidence of procedure-related, contrast-induced nephropathy (CIN). CIN is commonly defined as a decline in kidney function occurring in a narrow time window after administration of iodinated contrast agents. Although self-limiting in most cases, CIN carries a risk of more permanent renal insufficiency, dialysis, and death. It remains a common and serious complication among at-risk patients after exposure of contrast agents. Therefore, it is important to identify patients who are at risk during early stages to implement preventative strategies to decrease the incidence of CIN. Minimizing the amount of contrast administered and providing adequate hydration are the cornerstones of an effective preventative approach. This review focuses on the basic concepts of CIN and summarizes the current understanding of its pathophysiology. In addition, it provides practical recommendations with respect to CIN prevention and management.

## Introduction and background

Contrast media (CM) is given to patients before certain radiology imaging examinations to highlight features in the image to aid diagnosis. These include its use in computed tomography (CT) and magnetic resonance imaging (MRI). Depending on the nature of the condition being evaluated, CM may be administered orally, intravenously, or intra-arterially [1]. For example, in a barium swallow study, oral administration is predominant [1], whereas with a CT angiogram, intravenous iodine is used [2]. Although the type of CM varies, the most frequently used agents are iodine-based and gadolinium, a heavy metal CM used in MRI [2]. A complication associated with CM use is contrast-induced nephropathy (CIN) defined as a serious new-onset complication or exacerbation of renal dysfunction after administration of CM [1]. Laboratory tests indicating CIN include a >25% increase in serum creatinine (Scr) or an increase of ≥0.5 mg/dL from pre-administration values within 24-48 hours post-administration [3]. CIN is reported to be the third most common cause of hospital-acquired acute renal failure [4]. The incidence of CIN in patients who initially had normal renal function is less than 5% [5]. In patients with preexisting renal impairment, the prevalence is 12-27% [6]. Given the relationship between diabetes and its association with coronary disease, diabetic patients have become an important high-risk group for CIN [7]. CIN is associated with longer hospital stay, increased morbidity and mortality, and additional cost [8].

## Review

In this review, we attempted to answer the following questions: (1) What is the current evidence that CIN remains clinically relevant and a dangerous condition for the patient? (2) How do the physicochemical properties of CM play a role in CIN? (3) What is the evidence that periprocedural hydration is an effective, appropriate, and safe method to mitigate CIN?

Methods

The information in this review was obtained through a literature search of the following four electronic databases: PubMed, Biomedical Reference Collection Comprehensive, CINAHL Comprehensive, and EMBASE. A search was generated using the terms “contrast induced nephropathy” in the title in combination with the terms “contrast media,” “prevention,” and “diagnosis” within the text. The inclusion criteria were peer-reviewed published papers of human studies in English published between 1960 and 2020.

Effect on the kidney

CIN is the outcome of combined hypoxic and toxic renal parenchymal injury mediated by reactive oxygen species (ROS). Administration of CM decreases renal oxygenation in medullary structures, without reducing tubular reabsorption [9]. This occurs as the result of neurohumoral vasoconstrictive stimuli triggered by inhibition of nitric oxide (NO) and release of endothelin from endothelial cells exposed to CM [9]. Under normal physiological conditions, tubular transport is linked to ROS formation that occurs mostly in the renal medullary thick ascending limb (mTAL) [10]. It is within the mTAL where the dense mitochondrial population represents a major source for generation of superoxide anions and hydroxyl radicals by NADPH-oxidase [10].

Numerous studies have shown, either directly [11] or indirectly [12], that administration of CM enhances ROS production and renal oxidative stress, which, in turn, causes damage to cell membranes. This leads to cellular apoptosis and necrosis, particularly within the mTALs and in segments of proximal renal tubules of the outer medulla [13]. The administration of CM is linked to increased renal production of ROS metabolites such as malondialdehyde and F2 isoprostane which are markers of lipid peroxidation [13]. For example, clinical studies have shown that urinary excretion of F2 isoprostane was significantly increased in patients undergoing coronary angiography who had received CM [14]. Additionally, there is a two-fold rise in urinary 3-nitrotyrosine, a biomarker of reactive nitrogen species such as peroxynitrite. These events occurred instantly after coronary angiography with an intensity that depended on the volume of injected CM. This was interpreted as a process in which CM administration produces superoxide anions which causes formation of peroxynitrite through a chemical interaction with NO which then disables the NO-dependent vasodilatation. This then leads to a reduction of renal blood flow.

Risk factors

Preexisting renal dysfunction is the most significant risk factor for CIN, and up to 60% of the patients who develop CIN have a previous history of renal dysfunction [15]. In most patients, CIN is transitory, and baseline renal function returns to normal [15]. However, of those with chronic kidney disease, 56% progress to irreversible renal failure [16]. Additional risk factors include aging, female gender, congestive heart failure, dehydration, cirrhosis, nephrosis, atherosclerosis, and anemia [15]. Individuals with several risk factors are at a higher risk for developing CIN. There is a 5% increase in risk when there are two risk factors recognized which can increase to as much as 30% in patients with three risk factors [16]. Several risk factors for CIN have been noted in high-risk patients, mainly those with cardiovascular diseases, advanced age, hypertension, hyperuricemia, diabetes mellitus, and elevated C-reactive protein levels [17]. Other influences such as administration of numerous iodinated CM doses within a short time (within 24 hours), female gender, and the amount and type of the CM used play major roles [18].

Incidence of CIN in critically ill patients with stable renal function who underwent CT with intravenous CM has been evaluated and compared for patients <65 versus ≥65 years of age [19]. CIN has been defined as 25% or greater increase from baseline of Scr or as an absolute increase by 0.5 mg/dL until the fifth day after the infusion of contrast agent. Results indicated that critically ill patients ≥65 years of age were more likely to show renal injury after the intravenous infusion of CM. Therefore, critically ill elderly patients are more vulnerable to suffer from renal injury after intravenous contrast compared to younger patients [19].

Furthermore, the volume and type of CM used during angiography or coronary intervention also affect the occurrence of CIN. The administration of higher volume of CM and the use of concurrent nephrotoxic medication (nonsteroidal anti-inflammatory drugs, aminoglycosides, amphotericin B, high doses of loop diuretics, and antiviral drugs like acyclovir and foscarnet) medications are related to increased CIN and mortality [20,21]. Thus, reducing the volume of CM used and avoiding the use of nephrotoxic medications are obligatory prevention measures [22].

In addition, the physicochemical properties of the CM, such as its osmolality and viscosity, play a role in CM-related renal damage. Osmolarity indicates the concentration of particles in a solution and is expressed in osmoles of solute particles per liter solution (Osm/L). In the 1980s, Vernon Wallingford synthesized the first high-osmolar ionic contrast known as sodium acetrizoate which was acknowledged to be nephrotoxic [23]. During the time that ionic CM was administered extensively, it became evident that CM could cause serious side effects. These were primarily related to CM having a higher density and viscosity than human serum causing fluid to shift from cells to vasculature and impeding renal hemodynamics [23]. This limitation eventually led to the invention of the first low-osmolar non-ionic CM known as metrizamine in 1968 by a Swedish radiologist, Torsten Almèn [24]. Iso-osmolar media are the latest form of contrast agents used as they have the same osmolality as human serum and are less nephrotoxic compared with other contrast agents [25].

The NEPHRIC study [26], a randomized prospective double-blind study conducted in five European countries (Germany, Spain, Denmark, Sweden, and France), compared nephrotoxicity of an iso-osmolar, dimeric, non-ionic contrast medium, iodixanol, with a low osmolar, non-ionic, monomeric contrast medium, iohexol. This study included 129 patients with stable diabetes and impaired renal function undergoing coronary or aortofemoral angiography.

Each patient was randomly assigned to one of the two CM which were placed in identical vials to warrant blinding. The primary endpoint was the greatest rise in the Scr between day zero (day of contrast administration) and day three. Secondary endpoints were the number of patients with a peak increase of at least 0.5 mg/dL. Among the 129 patients in the study, 64 received iodixanol and the remainder the iohexol. The results indicated that the iodixanol solution had a significantly smaller mean increase in Scr (0.13 mg/dL) compared to iohexol (0.55 mg/dL). Most importantly, it was noted that about 3% of those who received iodixanol had an increase in their creatinine of ≥0.5 mg/dL compared to 26% in the iohexol group. It was concluded that the iso-osmolar contrast was considerably less nephrotoxic with the odds of CIN being 11 times as high in the low osmolar group compared to the iso-osmolar group [26].

Cardiac angiography is a procedure that may be followed by acute renal dysfunction (ARD). Administration of intravenous CM significantly lowers the risk of CIN than intra-arterial CM, especially if the arterial injection is suprarenal [27]. Patients who have a history of chronic kidney disease are at a higher risk of CIN, in part because of decreased recovery ability. Although administration of CM does not always lead to CIN in patients who have a history of renal insufﬁciency, renal failure requiring dialysis or even death occur more often in patients with CM exposure than in those with no exposure [28].

Diagnosis of renal failure

The diagnosis of CIN is typically based on an absolute (≥0.5 mg/dL) or relative (≥25%) increase in Scr from baseline [29]. According to some studies [30,31], Scr is not a precise marker of renal function due to at least three reasons. First, creatinine undergoes secretion by renal tubular epithelium normally. Second, there may be a discrepancy in its production from muscles, and it may vary with weight, race, age, and sex. Therefore, daily changes in Scr do not necessarily reflect changes in kidney function in patients with renal disease. Third, there is a dilution of creatinine during volume overload that may occur in patients with renal disease [31].

Because of these possible limitations of using only creatinine, numerous biomarkers of tubular injury have been considered. One such marker is plasma cystatin-C (Cys-C) which is an endogenous low-molecular-weight protease inhibitor that is easily filtered across the glomerular membrane and is neither secreted nor reabsorbed along the nephron. Cys-C is distributed in the extracellular fluid volume [32], while Scr is circulated in the total body water [33], a volume which is three times greater. Therefore, serum Cys-C increases more quickly than Scr when glomerular filtration rate (GFR) drops. During renal injury, a decrease in GFR leads to an increase in serum Cys-C, which occurs much earlier than the increase in Scr [34]. Due to its short half-life, Cys-C shows earlier changes in serum levels in comparison to Scr. Thus, making it a better marker than Scr as an indicator of GFR [34].

Another potential early marker of renal dysfunction is plasma neutrophil gelatinase-associated lipocalin (NGAL). NGAL is a protein related to human neutrophil gelatinase that belongs to the superfamily of lipocalins, which are small extracellular proteins serving to transport small hydrophobic molecules, such as lipids, pheromones, and retinoids [35]. Subsequent to renal tubular cell damage, NGAL is released into the plasma and urine; and is therefore increased in both urine and plasma much sooner (within two hours) than Scr [36]. In a prospective study by Wagener et al. [37] involving 81 adult patients undergoing cardiac surgery at Columbia University Medical Center of New York, 16 (20%) developed postoperative ARD. It was revealed that patients developing postoperative ARD had considerably higher urinary NGAL concentrations soon after cardiac surgery. These findings may improve the early detection of CIN and prevent progression to renal failure.

An additional marker of renal dysfunction is urinary liver-type fatty acid-binding protein (L-FABP) which is expressed in renal proximal tubules, secreted into urine, and is caused by decreased peritubular capillary blood flow and associated cellular hypoxia. The main role of FABP is to facilitate lipid transport in the renal tubules to the mitochondria or peroxisomes where they are metabolized by β-oxidation [38]. Experimental studies have revealed that urinary L-FABP has been elevated a few hours after cardiopulmonary bypass surgery, appearing as an independent risk indicator of ARD post-cardiac surgery, suggesting it may be a predictive early biomarker of ARD after cardiac surgery [39]. In an additional experiment, Bachorzewska-Gajewska et al. [36] studied 25 patients who had normal Scr and underwent percutaneous coronary intervention for unstable angina. It was shown that there was a significant increase in urinary L-FABP just after four hours and remained elevated for up to 48 hours [36].

Prevention strategies

Intravenous fluid hydration with normal saline is a low cost and low-risk fluid therapy found to be one of the main prevention strategies for CIN. Hydration increases intravascular volume, dilutes overall intravascular contrast load, and promotes diuresis [40]. Most importantly, hydration decreases the activity of renin-angiotensin system causing a reduction in vasoconstrictive substances, such as endothelin mitigating blood flow reduction and reducing cellular hypoxia [41]. It has been reported that the most effective method for reducing CIN risk is via hydration with intravenous isotonic saline prior to the administration of contrast [42]. In a randomized control study, it was shown that diabetic patients, patients receiving more than 250 mL of CM, female patients, and those underdoing emergent interventions were most likely to benefit from intravenous saline [43]. In this study, patients scheduled for elective or emergency coronary angioplasty benefited more from isotonic (0.9% saline) than half-isotonic (0.45% sodium chloride plus 5% glucose) beginning the morning of the procedure for elective interventions and immediately before emergency interventions.

Ascorbic acid (vitamin C) is a well-tolerated and readily available water-soluble antioxidant, which has been shown to diminish renal damage in a CIN rat model [44]. Aside from scavenging oxygen free radicals that arbitrate cell necrosis following myocardial infarction and angioplasty, ascorbic acid can likewise act as an antioxidant to prevent ischemic cell death in the renal tubule [45]. A randomized, double-blind study reported that prophylactic oral administration of the antioxidant ascorbic acid (3 g at least two hours before the procedure and 2 g in the night and the morning after the procedure) appears to reduce the incidence of CIN in patients with preexisting renal dysfunction undergoing coronary procedures [46].

An alternative potential preventative measure is the use of N-acetyl cysteine (NAC). NAC is a stable form of the non-essential amino acid cysteine that acts as a protective agent of the renal tubules. It is fairly inexpensive, readily available, has an ease of administration by both oral and intravenous injection, and is well tolerated in patients [47]. One of the contributing factors often associated with CIN is vasoconstriction. By stabilizing NO, NAC may have a vasodilatory effect in certain situations [48]. For example, it was shown in an animal study that pretreatment with NAC enhances renal blood flow by direct renal vasodilation and by releasing renal prostaglandin E2 and renal cortical NO [49]. In a double-blind study [50], 83 patients with an Scr concentration of >1.2 mg/dL or creatinine clearance of <50 mL/min undergoing CT scanning were studied. Patients received NAC (600 mg orally twice daily) and 0.45% saline intravenously (before and after CM administration), or a placebo and saline. In patients receiving NAC, Scr assessed 48 hours after CM administration decreased from its baseline value of 2.5 ± 1.3 to 2.1 ± 1.3 mg/dL (P < 0.001). In the placebo group, baseline Scr values (2.4 ± 1.3 mg/dL) did not decrease at 48 hours (2.6 ± 1.5 mg/dL). This suggests that prophylactic oral administration of NAC prior to contrast exposure may help prevent renal damage.

## Conclusions

In conclusion, CIN is a serious complication of angiographic procedures resulting from the administration of CM. The available literature has consistently shown that it is also considered a common problem resulting from interventional vascular procedures and is associated with a rise in morbidity, mortality, and increased length of hospital stay. It is possible that CIN is initiated soon after CM administration and that early and specific biomarkers could allow an early diagnosis of ARD and hopefully improve the patients’ outcome. As such, patients with CIN should be carefully monitored during their hospital stay and after discharge. More carefully designed studies are needed to fully elucidate the relationship between CIN and mortality. Studies suggest that intravenous hydration and antioxidant use prior to the procedure and using lessor amounts of CM are effective strategies toward preventing CIN. In this context, appropriate observation of renal function, identification of patients with risk factors, and management of successful preventive measures are of clinical importance. The goal of prevention is to protect the renal tubules from prolonged contact with CM by avoiding its use.
